# The Borderland of Madness

**Published:** 1889-10-12

**Authors:** P. H. Good

**Affiliations:** Chaplain Portsmouth Lunatic Asylum


### The Borderland of Madness.

By Rev. P. H. Good, M.A., Chaplain Portsmouth Lunatic Asylum.

Those who have to do with the insane must often have noticed how frequently allied mental delusions are to ordinary hallucinations, to which all are more or less liable. Everyone is conscious of the intermediate condition between sleeping and waking—in which the hallucinations and ideas of a dream struggle for a short time with returning consciousness—in which state the sleeper will either completely awake or drop off into another slumber. So Spinoza tells us of seeing (I suppose rather imagining he saw) a leprous negro on awaking out of sleep, of which he had just been dreaming, which image faded away when thoroughly awake. Aristotle gives instances of the same sort of thing in his treatise on "Dreams." Most persons can remember similar experiences.

A very grave and serious question arises here—I mean the question of responsibility. In this borderland of madness how far are people responsible for what they do? There are well authenticated cases in which persons who have been suddenly aroused from sleep have attacked those who awakened them, imagining they were face to face with some imaginary enemy of whom they had been dreaming. And a case is recorded of a sleeper being suddenly aroused, killing his wife.

A constable once gave evidence of meeting a woman in her nightdress coming out of a house. She cried out repeatedly, "Where is my child? I have thrown it out of window." She had just dreamed the house was on fire, and in her excitement in waking up snatched up her youngest and threw it out of window to save it from the flames, as she thought.

These are extreme cases, it is true, of excitement caused by dreams; and it may be a question in some of these instances whether an overlooked epileptic attack of a mild form might not have preceded the confusion and excitement of the half-waking delirium.

What is very solemn, and what ought to be borne in mind, is the problem, How far ought moral responsibility to be attributed to those who commit violence in a state of semi-consciousness arising from the excitement of some distressing dream?—[This is referred to elsewhere.—Ed.]

